# Beyond PET/CT: the evolving role of circulating tumor DNA in follicular lymphoma

**DOI:** 10.3389/fimmu.2026.1978141

**Published:** 2026-10-27

**Authors:** Santiago Barrio, Alejandro Martín-Muñoz, Ana Jiménez-Ubieto

**Affiliations:** 1 Medical Oncology Department, Hospital Universitario Puerta de Hierro-Majadahonda, Instituto de Investigación Sanitaria Puerta de Hierro-Segovia de Arana (IDIPHISA), Madrid, Spain; 2 Altum Sequencing S.L., Madrid, Spain; 3 Hematology Department, Hospital Universitario 12 de Octubre, Madrid, Spain

**Keywords:** circulating tumor DNA, follicular lymphoma, minimal residual disease, molecular monitoring, PET/CT

## Introduction

Response assessment in follicular lymphoma (FL) has traditionally relied on imaging. The introduction of ^18^F-FDG PET/CT represented a major advance over anatomical assessment and established end-of-treatment metabolic response as a powerful prognostic marker in FL (1, 2). Nevertheless, PET remains an indirect measure of lymphoma biology. FDG uptake may reflect inflammation, immune activation, or treatment-related changes, whereas low-volume residual disease may remain below the spatial resolution of imaging.

These limitations are particularly relevant in FL, a disease characterized by a prolonged and heterogeneous natural history. Circulating tumor DNA (ctDNA) offers a complementary approach by detecting tumor-derived molecular material through a minimally invasive plasma sample. Unlike a single-site biopsy, ctDNA may also reflect disease arising from multiple anatomical locations, potentially providing a broader representation of tumor heterogeneity (3).

PET/CT and ctDNA should therefore not be considered competing biomarkers. They assess different dimensions of disease—metabolic activity and molecular tumor burden—and discordant findings may be informative rather than contradictory. This complementarity was supported by our initial real-world study, in which individualized ultra-deep ctDNA sequencing provided prognostic information during first-line treatment and complemented PET/CT response assessment (4).

At this stage, three questions should be kept distinct: whether ctDNA can be measured reliably, whether the result predicts outcome, and whether acting on that result improves patient care. In FL, evidence is advancing rapidly for analytical and prognostic validity, whereas clinical utility remains largely unproven. Our view is that ctDNA is sufficiently mature for prospective incorporation into multimodal response assessment and clinical-trial design, but current evidence does not support its use as a stand-alone trigger for treatment initiation, escalation, or de-escalation outside a clinical trial.

## Expanding MRD assessment in follicular lymphoma

Molecular monitoring in FL predates ctDNA. Earlier approaches mainly relied on PCR detection of the BCL2::IGH rearrangement and established the prognostic relevance of molecular remission. Their clinical applicability, however, was limited by the absence of an informative molecular marker in a substantial proportion of patients and by reliance on a single genomic alteration.

Next-generation sequencing has substantially expanded this approach. Tumor-informed strategies can identify several patient-specific somatic alterations and subsequently monitor them in plasma, increasing both the applicability and biological breadth of molecular assessment.

Unlike hematologic malignancies in which bone marrow-based MRD assessment is well established, FL presents distinct challenges due to its predominantly nodal distribution, indolent course, and variable ctDNA shedding, underscoring the need for disease-specific validation.

Important biological and technical limitations remain. ctDNA shedding in FL may be low, particularly in patients with low-volume or predominantly nodal disease, and varies with tumor burden and anatomical distribution. Assay performance also depends on plasma volume, pre-analytical handling, sequencing depth, error suppression, and the number and type of alterations monitored. Consequently, an undetectable ctDNA result does not necessarily exclude residual disease and should be interpreted in relation to the assay used, the clinical setting, and contemporaneous imaging (13, 14).

Several studies now provide quantitative support for the prognostic relevance of ctDNA during first-line therapy in FL. In the prospective study by Fernández-Miranda et al., which included 100 plasma samples from 36 patients, pretreatment ctDNA levels were higher in patients who did not achieve complete response (P = 0.02) and in those with POD24 (P < 0.001) (5). In our real-world study, 84 patients were screened and 151 follow-up plasma samples from 54 treated patients were analyzed. Positive LiqBio-MRD during first-line therapy was associated with progression both at interim assessment (HR, 11.0; 95% CI, 2.10–57.7; P = 0.005) and at end of treatment (HR, 19.1; 95% CI, 4.10–89.4; P < 0.001); combining LiqBio-MRD with PET/CT identified POD24 with 88% sensitivity and 100% specificity (4).

More recently, Claudel et al. evaluated end-of-induction ctDNA together with PET in 141 patients receiving first-line therapy (6). Using phased variants, ctDNA was detected at baseline in 140 patients (99.3%). Median progression-free survival was not reached in the 112 patients with undetectable ctDNA at end of induction, compared with 17.7 months in ctDNA-positive patients. For POD24, positive predictive value was 58.3% for ctDNA and 45.0% for PET, increasing to 85.7% when both were positive, while negative predictive value remained above 90%. These findings quantify the complementary rather than interchangeable contribution of molecular and metabolic response.

ctDNA technologies should not be considered interchangeable. Conventional mutation-based panels can be applied without a patient-specific assay but may have lower sensitivity at very low disease burden. Tumor-informed ultra-deep sequencing requires suitable tumor material and assay design but enables monitoring of multiple patient-specific variants. Phased-variant approaches track two or more mutations on the same DNA fragment to improve error suppression and sensitivity (7). Platforms also differ in plasma and tissue requirements, genomic breadth, sequencing depth, reporting units, and definitions of molecular positivity. Results, thresholds, and sampling landmarks validated for one platform therefore cannot be assumed to apply to another (13, 14).

Increasing analytical sensitivity also creates a clinical challenge: detecting very small amounts of tumor-derived DNA does not by itself determine what should be done for an individual patient. In an indolent lymphoma such as FL, biological significance and clinical actionability must be distinguished.

Discordant PET/CT and ctDNA results deserve particular attention. PET-positive/ctDNA-negative findings may reflect inflammation or treatment-related change, but may also represent residual disease with insufficient ctDNA shedding or below the analytical sensitivity of the assay. PET-negative/ctDNA-positive findings may indicate molecular persistence below imaging resolution. Their meaning depends on timing, treatment, disease distribution, and assay characteristics. Neither state should currently be interpreted in isolation as a mandate to escalate or de-escalate therapy; prospective studies should predefine how discordance is adjudicated and assess serial molecular trajectories rather than a single landmark alone.

## What did FOLL12 teach us?

This distinction is particularly well illustrated by the FOLL12 trial (8). FOLL12 prospectively compared standard rituximab maintenance with a response-adapted post-induction strategy based on PET and conventional molecular MRD. Although this approach differed substantially from contemporary tumor-informed ctDNA strategies, the response-adapted strategy did not improve outcome compared with standard maintenance.

FOLL12 therefore provides an essential lesson for the development of ctDNA in FL: a prognostic biomarker is not necessarily an actionable biomarker. Demonstrating that a molecular measurement identifies patients with different outcomes does not establish that modifying treatment according to that result will improve survival or disease control.

FOLL12 should not be regarded as evidence against molecular monitoring. Rather, it highlights the challenge of translating molecular information into treatment decisions. Newer tumor-informed approaches can expand the proportion of evaluable patients and improve analytical sensitivity, but their clinical utility must ultimately be demonstrated prospectively. This is especially important in FL, where treatment initiated solely because of molecular positivity could expose an asymptomatic patient to unnecessary therapy.

## ctDNA in the era of cellular and bispecific therapies

The expanding therapeutic landscape of relapsed/refractory FL provides another setting in which ctDNA may be particularly informative. CAR-T cells and bispecific antibodies produce deep responses through mechanisms that differ substantially from conventional chemoimmunotherapy. Following CAR-T cell therapy and bispecific antibody treatment, immune activation and inflammatory responses may result in increased FDG uptake even in the absence of viable lymphoma, complicating PET/CT interpretation. In this setting, ctDNA may provide complementary molecular information to help distinguish residual disease from treatment-related inflammatory changes, although this approach requires prospective validation. However, the available ctDNA evidence after these therapies remains exploratory, derives from relatively small or selected cohorts, and does not yet establish a management strategy. The findings below should therefore be viewed as hypothesis-generating observations that require treatment-specific validation (13, 14).

In our proof-of-principle study of 11 patients treated with anti-CD19 CAR-T cells, 59 serial plasma samples were analyzed over a median follow-up of 36 months (9). All 14 PET/CT-negative assessments with paired molecular testing were ctDNA-negative. All durably responding patients had undetectable ctDNA at or around three months, whereas both patients who progressed were LiqBio-MRD-positive before or at progression. Two PET-positive/ctDNA-negative discordances were not followed by confirmed progression, illustrating how ctDNA may help interpret equivocal post-immunotherapy imaging.

Bispecific-antibody data reinforce this concept. In the ELM-2 FL cohort treated with odronextamab, 32 of 60 patients (53.3%) had undetectable ctDNA at cycle 4 day 15. Their median progression-free survival was 42.4 months, compared with 21.6 months in patients with detectable ctDNA (HR, 0.31; 95% CI, 0.14–0.67) (10). Even among patients in radiographic complete response, undetectable ctDNA identified a group with longer progression-free survival (42.4 vs 22.0 months; HR, 0.35; 95% CI, 0.15–0.84). Consistent with these findings, 77 of 128 patients with relapsed/refractory FL treated with epcoritamab had evaluable MRD data; 49 of 77 (64%) had undetectable ctDNA at cycle 3 day 1, which was associated with longer progression-free survival (12). Together with analyses of response dynamics after odronextamab (11), these data suggest that radiographic complete response is not a biologically homogeneous state.

The timing of molecular assessment is likely to depend on treatment mechanism. In our CAR-T cohort, none of the patients tested at day +7 had cleared ctDNA, including six with durable responses (9), whereas later landmarks were informative. Thus, thresholds and sampling landmarks developed during chemoimmunotherapy should not automatically be transferred to CAR-T cells or bispecific antibodies. Treatment-specific validation will be essential if molecular response is to become a reproducible clinical endpoint.

Representative quantitative results across first-line therapy, CAR-T therapy, and bispecific-antibody treatment are summarized in **Table 1**.

**Table 1:** Selected quantitative studies of ctDNA monitoring in follicular lymphoma.

Study	Setting	Patients / samples	Key quantitative findings
Fernández-Miranda et al. (5)	First-line FL	36 patients; 100 plasma samples	Higher baseline ctDNA in non-CR and POD24 patients
Jiménez-Ubieto et al. (4)	First-line FL; PET + ctDNA	84 screened; 54 monitored	Interim MRD+: HR 11.0; EOT MRD+: HR 19.1
Claudel et al. (6)	First-line FL; PET + ctDNA	141 patients	EOI ctDNA+: median PFS 17.7 months vs NR; improved POD24 prediction with PET + ctDNA
Jiménez-Ubieto et al. (9)	CD19 CAR-T; proof of concept	11 patients; 59 samples	14/14 PET-negative assessments were ctDNA-negative
Luminari / Jiménez-Ubieto et al., ELM-2 (2026) (11)	Odronextamab; R/R FL	53 patients in CR with evaluable ctDNA	Week-12 ctDNA undetected vs detected: median PFS NR vs 21.6 months; HR 0.30
Altıntaş et al., EPCORE NHL-1 (2026) (12)	Epcoritamab; R/R FL	128 patients	At C3D1, 49/77 (64%) had undetectable ctDNA; MRD negativity associated with longer PFS
Avilion et al., TRANSCEND FL (ASCO 2026) (15)	Liso-cel CAR-T; PhasED-Seq	80 evaluable patients	MRD−: 89%; 36-month PFS 80% vs 38% (MRD− vs MRD+)

C3D1, cycle 3 day 1; C4D15, cycle 4 day 15; CR, complete response; EOI, end of induction; EOT, end of treatment; MRD, measurable residual disease; NPV, negative predictive value; PFS, progression-free survival; POD24, progression of disease within 24 months; PPV, positive predictive value; R/R, relapsed/refractory.

## From prognostic value to clinical utility

The evidence now supports ctDNA as a prognostic biomarker across first-line chemoimmunotherapy, CAR-T therapy and bispecific antibodies. The remaining question is whether molecular information can improve clinical management.

Prospective trials should specify what ctDNA is intended to change: intensifying or de-escalating treatment, shortening or extending therapy, prompting additional imaging or biopsy, or initiating pre-emptive treatment at molecular recurrence. Ultimately, clinical utility should be established in prospective randomized trials comparing standard management, with ctDNA results blinded to treating clinicians, against a prespecified ctDNA-guided management strategy. These trials should evaluate whether molecularly guided decisions improve clinically meaningful outcomes, including progression-free survival, treatment-related toxicity, and quality of life, while generating evidence to inform future coverage and reimbursement decisions. A ctDNA-guided strategy will be meaningful only if it changes management in a way that improves a patient-relevant outcome.

The costs of ctDNA testing, particularly for personalized ultra-deep sequencing and serial monitoring, represent an additional consideration for its implementation in routine clinical practice. Future prospective trials should incorporate health-economic endpoints to determine whether ctDNA-guided strategies are cost-effective, taking into account assay costs, resource utilization, and the potential avoidance of unnecessary treatments and investigations.

This issue is especially important for molecular relapse. Detecting ctDNA before clinical or radiographic progression may increase lead time, but earlier detection is not itself evidence of clinical benefit. In an indolent disease, some patients may remain asymptomatic for a prolonged period, and intervention at molecular relapse could lead to additional imaging or biopsies, anxiety, overdiagnosis, and premature treatment without improving survival or quality of life. Routine surveillance or pre-emptive treatment based on ctDNA should therefore await prospective trials demonstrating that acting on molecular relapse improves meaningful outcomes (14).

Until such evidence is available, ctDNA should complement rather than replace established clinical, pathological and imaging assessment. Tissue biopsy remains essential at diagnosis and whenever transformation is suspected, while PET/CT provides unique anatomical and metabolic information. ctDNA adds a minimally invasive, repeatable measure of molecular burden and may be particularly valuable in apparently complete responses or discordant cases.

In our view, ctDNA should not replace PET/CT in FL, but it is ready to be prospectively evaluated as a complementary biomarker at clinically defined timepoints. Its greatest immediate value may lie in refining the interpretation of equivocal imaging findings and identifying patients who require closer assessment after treatment. However, ctDNA-directed surveillance or therapeutic intervention should remain investigational until prospective trials demonstrate that acting on molecular findings improves patient outcomes.
